# Bridging the gap to meet complex needs: an intersectoral action well supported by appropriate policies and governance

**DOI:** 10.1186/s12961-024-01171-1

**Published:** 2024-07-03

**Authors:** Catherine Hudon

**Affiliations:** Department of Family Medicine and Emergency Medicine, Université de Sherbrooke, Pavillon Z7-local 3007, 3001, 12E Avenue Nord, Sherbrooke, QC J1H 5N4 Canada

**Keywords:** Social determinants of health, Health equity, Integrated care, Complex needs

## Abstract

Many people face problems about physical, mental, and social dimensions of health, and may have complex needs. They often experience a mismatch between their needs and the ability of the healthcare system to meet them, resulting in under- or overutilization of the healthcare system. On one hand, improving access to community-based primary healthcare for hard-to-reach populations should bring all healthcare and social services to one point of contact, near the community. On the other hand, better addressing the unmet needs of people who overuse healthcare services calls for integrated care among providers across all settings and sectors. In either case, intersectoral action between healthcare and social professionals and resources remains central to bringing care closer to the people and the community, enhancing equitable access, and improving health status. However, efforts to implement integrated care are unevenly weighted toward clinical and professional strategies (micro level), which could jeopardize our ability to implement and sustain integrated care. The development of appropriate policies and governance mechanisms (macro level) is essential to break down silos, promote a coherent intersectoral action, and improve health equity.

Mrs. Martin (fictitious name), a 52-year-old woman, lives with a spouse with an alcohol problem who becomes physically violent when he drinks. She is socially isolated and receives minimal income from the government. She suffers from many physical illnesses as well as from anxiety. Facing this kind of reality, many people like Mrs. Martin will take one of two opposite paths: under- or overusing the healthcare system.

Living in contexts of socioeconomic precariousness significantly increases the risk of developing mental health and physical illnesses [1]. Thus, an increasing number of people face interacting challenges among the physical, mental, and social dimensions of health. These people form a variety of complex needs profiles either for their age, personal characteristics, or health conditions. Despite this great heterogeneity, one important characteristic shared by most of them remains the gap between their needs and the capacity of the healthcare system to meet them [2]. Unfortunately, as per the inverse care law, increased complexity of people’s needs comes with decreased care accessibility, putting these people at increased risk of poorer outcomes [3]. The greater the gap, the more complex their needs become, and the worse their health is likely to be, producing a vicious cycle of more complex needs and worse health.

For some populations (e.g., newcomers, people in remote locations, homeless people), barriers hinder their capacity to access the healthcare system [4]. Communication (language barrier, low literacy, difficulty accessing information, etc.) and organizational barriers to access (long waiting lists, lack of resources, difficult access, etc.) may lead to isolation and disengagement in care. While this could be the case for Mrs. Martin, she might also try to meet her unmet needs by repeatedly visiting the emergency room and other services [5]. High use of healthcare services may lead to uncoordinated and fragmented care. Not only do they often have bad experiences and poor health outcomes, but the lack of efficiency also generates dissatisfaction among providers and high costs for the healthcare system [6].

## Adapted intersectoral strategies to better meet the needs of these people

Rather than assuming that a situation like Mrs. Martin’s is outside of our control, it is the responsibility of an equitable healthcare system to proactively deploy strategies to better meet her needs, regardless of whether she under- or overuses healthcare services. However, we must keep in mind that each situation (under- or overuse) calls for an adapted solution.

On one hand, improving access to community-based primary healthcare for hard-to-reach populations should bring all healthcare and social services to one point of contact, near the community [7]. The services should be adapted to the specific needs of the targeted population and must include key ingredients such as adaptability, flexibility, and relationship building [4]. Mrs. Martin could therefore receive all the care she needs, including support for women who are victims of violence, through an easily accessible resource close to her home.

On the other hand, better addressing the unmet needs of people who overuse healthcare services calls for integrated care [8] among providers across all settings and sectors. According to the World Health Organization, integrated care should be deployed from the user’s perspective. Care should be planned and coordinated, with all partners, around the needs of the people and their family, while putting them in control. From that perspective, any intervention aiming to improve integrated care (e.g., case management) should include strategies to evaluate and understand people’s reality and needs, putting their life project and motivations at the heart of the intervention, and coordinating care across settings and sectors (e.g., an individualized service plan developed with the person, their family, and all intersectoral partners). Including self-management support strategies such as motivational interviewing, for example, may help people develop their own capacity and regain control. If Mrs. Martin frequently used healthcare services, she could meet regularly, and build a trusting relationship, with a case manager who would develop an individualized service plan and coordinate her care around her life project, with all partners, including a social worker and, ideally, a community resource that is familiar with domestic violence.

## An intersectoral action well supported by appropriate policies and governance

In either case (under- or overuse), integrated care between healthcare and social professionals and resources (e.g., social workers and community organizations) remains central to bringing care closer to the people and the community, enhancing equitable access, and improving health status [9]. All these actors have a collective responsibility to better meet the needs of people with complex needs.

However, efforts to implement integrated care are unevenly weighted towards clinical and professional strategies (micro level) [10]. The inconsistency or lack of appropriate policies or governance mechanisms (macro level) to support and guide micro-level strategies can jeopardize our ability to implement and sustain integrated care. Therefore, a rebalance is essential to break down silos, promote a coherent intersectoral action, and improve equity in health.

## Macro-level strategies to impact socioeconomic determinants of health upstream

Not only does intersectoral action play a central role, it must go beyond the healthcare system to try reducing the impact of socioeconomic determinants of health upstream [11]. The earlier in life that we collectively succeed in improving these determinants for those who are most vulnerable, the greater the impact will be on health by reducing the occurrence of mental and physical illnesses; thus, curbing the development of complex needs.

Promoting education, ensuring minimal income, breaking isolation, preventing violence, and improving people’s living environment require concerted strategies between several sectors (health, social services, education, etc.). Once again, we must remain vigilant to avoid weighting unevenly toward micro-level actions. To achieve success in addressing such a societal challenge, it is necessary to implement cross-sectoral policy integration strategies.

## Data Availability

Not applicable.
